# Draft genome sequences data of rare *Salmonella enterica* sub sp. *enterica* serovar Ceyco and serovar Hillegersberg isolated from diarrheal patients in India

**DOI:** 10.1016/j.dib.2022.107875

**Published:** 2022-01-26

**Authors:** Jobin John Jacob, Tharani Priya T, Dhanalakshmi Solaimalai, Yesudoss M, Jansi Rani Malaiyappan, Tanya Rachel, Aravind V, Monisha Priya T, Praveena Jeslin, Biju George, Balaji Veeraraghavan

**Affiliations:** a Department of Clinical Microbiology, Christian Medical College, Vellore, Tamil Nadu; b Department of Haematology, Christian Medical College, Vellore Tamil Nadu, India

**Keywords:** Non-Typhoidal *Salmonella*, Whole genome sequencing, Genomics, Diarrhea, India

## Abstract

We report here the draft genome sequence of two rare *Salmonella* serotypes, isolated from human faecal samples in India. The isolates were identified as *Salmonella enterica* subsp*. enterica* serovar Ceyco and serovar Hillegersberg by Wole genome sequencing (WGS) based serotype prediction. The genomic similarity of study isolates was identified by clustering with the global collection of *Salmonella* sp. available in EnteroBase and SISTR based on their cgMLST profile. Phylogenetic analysis showed the study isolates were closer to *S*. Detmold and other unknown serovars from serogroup D_2__._ The information generated from genome sequencing of two rare *S. enterica* serovar will improve the overall understanding of the epidemiology of this clinically relevant pathogen.

## Specifications Table

**Table:** 

Subject	Biological Sciences
Specific subject area	Clinical Microbiology; Bacterial genomics
Type of data	Genome sequence data, table, figure
How the data were acquired	Whole genome sequencing: Illumina iSeq-100 De novo assembly: SPAdes v3.15.3 Phylogenetic analysis: Mash tree v.0.30 Tree visualization: iTOL v6
Data format	Raw paired-end Illumina sequences assembled and annotated
Description of data collection	The clinical isolates were cultured on Xylose Lysine Deoxycholate (XLD) agar and the Genomic DNA was isolated using Wizard DNA purification kit for Whole genome sequencing. The Raw reads were assembled using SPAdes and annotated by NCBI Prokaryotic Genome Annotation Pipeline (PGAP v. 4.1)
Data source location	*Salmonella* strains were isolated from patients admitted at Christian Medical College Hospital, Vellore, India (12.9256 N 79.1359 E)
Data accessibility	The Raw reads and assembled genome sequences are deposited in GenBank under the BioProject number PRJNA692535 and PRJNA767943. Repository name: NCBI Sequence Read Archive (SRA) Data identification number: PRJNA692535, PRJNA767943 Direct link to the data: https://sra-pub-run-odp.s3.amazonaws.com/sra/SRR16229856/SRR16229856 https://sra-pub-run-odp.s3.amazonaws.com/sra/SRR16474915/SRR16474915

## Value of the Data

• The availability of genome sequencing data of rare *Salmonella* sp. provides insight on genetic diversity of the species
• The data also helps to understand the genomic epidemiology of this clinical pathogen
• The data can be used to identify other untypable *Salmonella* serotypes based on the genomic similarity and antigenic formulae

## 1 Data Description

*Salmonella enterica* subsp*. enterica* is one of the major causes of bacterial diarrhea across the world. Based on the antigenic variations (O, H1, H2 and Vi) *Salmonella enterica* is classified into >2,500 serotypes. Serovar determination by phenotypic characterization of the O and H-antigens of *Salmonella* by the slide agglutination test, often generate untypable serovar designation. Therefore, seven-gene MLST based molecular subtyping has been commonly employed to accurately infer *Salmonella* serovar designations. Unfortunately, Multilocus sequence typing (MLST) does not differentiate all serotypes (Eg. polyphyletic serovars). Hence whole genome sequencing (WGS) has been recently used to comprehensively identify untypable or rare serovars. Here we report two untypable rare serovars belonging to *Salmonella enterica* sub sp. *enterica* serovar Ceyco and serovar Hillegersberg isolated from diarrheal patients in India.

*S.* Ceyco was first identified in the year 1966 from human samples in India and reported to have reappeared in the year 1969 [1]. Similarly, *S.* Hillegersberg was first reported from a patient in Municipal Health Laboratory, Rotterdam, Netherlands [2]. Both serovars are rarely isolated in most countries and have not been characterized from clinical samples since the preliminary identification reports. The study isolate, *S*. Ceyco strain FC2085 was recovered from the stool sample of a 7-year-old extramedullary leukemic relapse patient admitted at Christian Medical College, Vellore, India. The second isolate, *S.* Hillegersberg strain FC2223 was isolated from the stool sample of a 20-year-old man with Anaplastic large cells lymphoma. The strains were isolated as per standard microbiology techniques from stool samples and serogrouped as 9,46 (D_2_) with commercial typing antiserum based on Kauffman-White scheme [3]. Antimicrobial susceptibility testing was performed and both isolates were susceptible to tested antimicrobials except aminoglycosides. The breakpoints were interpreted according to Clinical and Laboratory Standards Institute guidelines [4].

## 2 Experimental Design, Materials and Methods

Genomic DNA was extracted from the overnight culture of the isolates using Wizard DNA purification kit (Promega. Madison, WI). Sequencing ready, paired end library was prepared using 100 ng of DNA with the Nextera DNA flex library prep kit (Illumina, Inc., San Diego, USA). This was followed by sequencing on Illumina iSeq-100 platform with a paired-end run of 2 × 150 bp. Trimmed reads were *de novo* assembled using SPAdes (v.3.15.3) with default settings (https://github.com/ablab/spades) which resulted in a coverage of 71x and 23x for *S.* Ceyco and *S.* Hillegersberg respectively. The draft genome was annotated using the NCBI Prokaryotic Genome Annotation Pipeline (PGAP v. 4.1) and subsequently deposited at GenBank.

The raw sequencing reads of strain FC2085 and FC2223 were submitted to SeqSero (v.2.0) [5] to determine the antigenic formula to predict the serotype. Strain FC2085 was identified as *Salmonella enterica* serovar Ceyco with the antigenic formula ‘9,46:k:z35’. Notably, strain FC2223 was predicted to be ‘9,46:a:z35’ (novel) as per SeqSero2. However, the antigenic formula was later confirmed as ‘9,46:z35:1,5’ that belongs to *Salmonella enterica* serovar Hillegersberg upon analysis by the Centre for Reference and Research on *Salmonella*, Pasteur Institute in Paris, France. The assembled genome size of *S.* Ceyco strain FC2085 was 4,691,294 bp with a G+C content of 51.8% and N50 value of 417,690. Concurrently, *S.* Hillegersberg strain FC2223 accounted for a genome size of 4,744,996 bp with a G+C content of 52% and N50 value of 51,438. Gene prediction and annotation showed a total of 4,408 and 4,586 coding sequences for *S.* Ceyco and *S.* Hillegersberg respectively (Table 1).

**Table 1:** General genome characteristics of *S*. Ceyco strain FC2085 and *S*. Hillegersberg strain FC2223.

Features	FC2085	FC2223
Status	Draft	Draft
NCBI BioSample no.	SAMN17348777	SAMN21988699
SRA accession no.	SRR16229856	SRR16474915
GenBank accession no.	GCA_016745495.1	GCA_020551995.1
No. of contigs	37	177
Total length (bp)	4,691,294	4,744,996
Total No. of CDS	4,408	4,586
N50 (bp)	417,690	51,438
GC content (%)	51.8	52
Coverage (×)	71.0x	23.0x
No. of reads	778972	270818
Serogroup	D_2_	D_2_
Serotype	Ceyco	Hillegersberg
AMR genes	*aac(6′)-Iaa*	*aac(6′)-Iaa*
SPI	SPI-1, 2, 3, 9 & C63PI	SPI-1, 2, 3 & C63PI
CRISPR Arrays	2 Loci 1: 23 spacers Loci 2: 16 spacers	3 Loci 1: 40 spacers Loci 2: 10 spacers

MLST profile of isolates from genome assembly revealed new sequence types (ST) and STs were subsequently assigned as ST8445 for *S*. Ceyco strain FC2085 and ST8446 for *S*. Hillegersberg strain FC2223 (http://enterobase.warwick.ac.uk/species/senterica). Clustered regularly interspaced short palindromic repeat (CRISPR) typing of the study isolates identified using CRISPRDetect (http://crispr.otago.ac.nz/CRISPRDetect/predict_crispr_array.html) showed two CRISPR loci for both the isolates with loci 1 and 2 of strain FC2085 carrying 23 and 16 spacers respectively. Similarly, strain FC2223 carried 40 spacers in loci 1 and 10 in loci 2. Resistome analysis of study isolates using ResFinder v.4.1 (https://cge.cbs.dtu.dk/services/ResFinder/) showed only chromosomal-encoded *aac(6′)-Iaa* gene, which confers aminoglycoside resistance, and *parC* T57S point mutation. Plasmids were not detected in both the isolates as analyzed by PlasmidFinder (https://cge.cbs.dtu.dk/services/PlasmidFinder/).

**Fig. 1:**
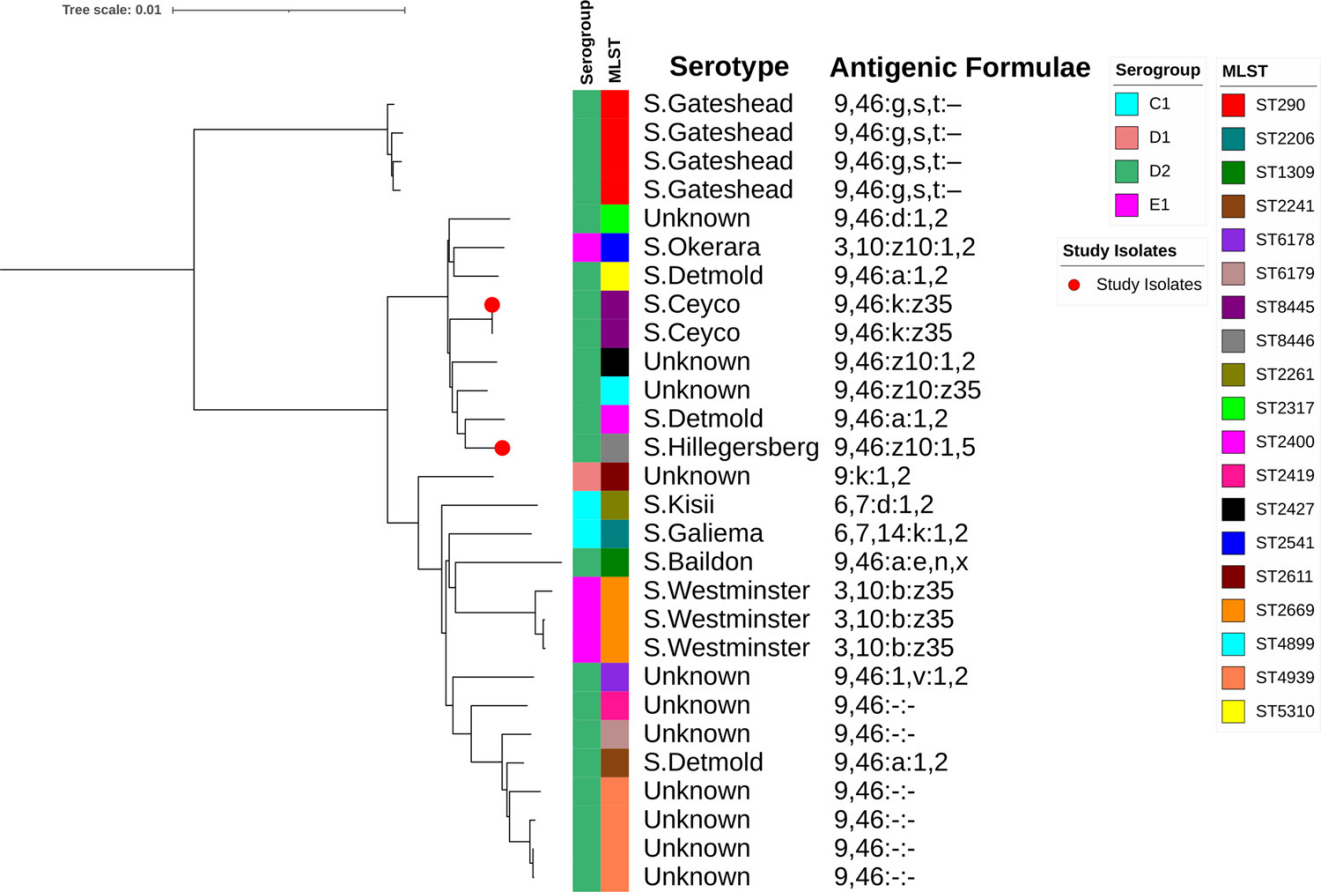
Genetic distance-based tree generated by Mash tree of *Salmonella* genomes grouped into the same clusters by both GrapeTree MLST and SISTR cgMLST analysis. The phylogenetic tree was annotated tree with Interactive Tree Of Life. Serogroups and MLST are displayed with colour strips.

The study isolates were placed in the global phylogenomic framework based on Core genome MLST (cgMLST) available in SISTR (https://lfz.corefacility.ca/sistr-app/?#) [6]. The dendrogram hence generated displayed the phylogenetic position of study isolates and closely related isolates were selected for further analysis. Similarly, isolates clustered with the isolates as per GrapeTree clustering were identified from EnteroBase [7]. Representative genomes, hence identified (*n=26*) were used to generate the phylogenetic tree using Mash tree (https://github.com/lskatz/mashtree) [8]. The resulting phylogenetic tree was visualised and annotated using the Interactive Tree of Life software (iTOL v6) [9]. Our isolates were found to be phylogenetically closer to *S.* Detmold and other unknown serovars from serogroup D_2_ (Fig. 1). The information generated from genome sequencing of two rare *S. enterica* serovar will improve the overall understanding of the epidemiology of this clinically relevant pathogen. The raw reads and assembled genome sequences of *S*. Ceyco strain FC2085 and *S*. Hillegersberg strain FC2223 have been deposited in GenBank under the Biosample number PRJNA224116 and PRJNA767943.

## Ethics Statements

The isolates used in this study were collected in the Clinical Microbiology Laboratory of the Christian Medical College and Hospital Vellore. No patients were recruited and the data collected from patients samples was anonymized and hence ethical approval and informed consent statements are not applicable. All prevailing local, national and international regulations and conventions and normal scientific ethical practices have been respected and all ethical norms have been followed. Ethical requirements in accordance with the World Medical Association was strictly followed.

## CRediT Author Statement

**Jobin John Jacob:** Conceptualization, Methodology, Data curation, Writing- Original draft preparation; **Tharani Priya T:** Methodology, Data curation Writing- Original draft preparation; **Dhanalakshmi Solaimalai:** Data curation, Supervision; **Yesudoss M:** Investigation, Data curation; **Jansi Rani Malaiyappan:** Visualization, Data curation; **Tanya Rachel:** Investigation, Data curation; **Aravind V:** Software, Validation; **Monisha Priya T:** Software, Validation; **Praveena Jeslin:** Investigation, Supervision; **Biju George:** Investigation, Supervision; **Balaji Veeraraghavan:** Conceptualization, Supervision, Writing- Reviewing and Editing.

## Declaration of Competing Interest

The authors declare that they have no known competing financial interests or personal relationships that could have appeared to influence the work reported in this paper.
